# A duplex, SYBR Green I-based RT-qPCR assay for the simultaneous detection of *Apple chlorotic leaf spot virus* and *Cherry green ring mottle virus* in peach

**DOI:** 10.1186/1743-422X-10-255

**Published:** 2013-08-10

**Authors:** Zhe Zhao, Yun Yu, Zhixiang Zhang, Pengbo Liang, Yuxin Ma, Shifang Li, Hongqing Wang

**Affiliations:** 1 College of Agronomy and Biotechnology, China Agricultural University, Yuanmingyuan West Road No. 2, Haidian District, Beijing 100193, People’s Republic of China; 2 State Key Laboratory of Biology of Plant Diseases and Insect Pests, Institute of Plant Protection, Chinese Academy of Agricultural Sciences, Yuanmingyuan West Road No. 2, Haidian District, Beijing 100193, People’s Republic of China

**Keywords:** ACLSV, CGRMV, SYBR Green I, Duplex real-time PCR, RT-qPCR

## Abstract

**Background:**

Co-infections of *Apple chlorotic leaf spot virus* (ACLSV) and *Cherry green ring mottle virus* (CGRMV) in peach is common in China and have resulted in significant yield reductions. A reliable, sensitive and quantitive method is needed to detect and distinguish between ACLSV and CGRMV in peach.

**Findings:**

We developed a sensitive and specific SYBR Green-I based RT-qPCR for the quantification of ACLSV and CGRMV in different peach tissues, and a duplex RT-qPCR system to detect ACLSV and CGRMV simultaneously. The RT-qPCR method was optimized using standard samples transcribed by the T7 Large Scale RNA Production System in vitro. The peach genes, *RNA Polymerase subunit II* (*RPII*) and *Ubiquitin 10* (*UBQ10*), which were used as the internal controls for the quantification assay also showed good expression stability in this system. Single RT-qPCR assays showed that CGRMV in peach accumulates to a higher level than ACLSV. The detection limits of the duplex RT-qPCR assay were 10^2^ and 10^4^ copies for ACLSV and CGRMV, respectively. The sensitivity of the duplex RT-qPCR was as high as RT-qPCR and higher than RT-PCR.

**Conclusions:**

The SYBR Green-I RT-qPCR assay provided a sensitive, specific and reliable method for the detection and quantification of ACLSV and CGRMV in different peach tissues. The duplex RT-qPCR system provided a sensitive and specific method to detect and differentiate between ACLSV and CGRMV in a single sample. This RT-qPCR assay could be a useful tool for the routine diagnosis of these two viruses and for disease epidemiology studies in peach orchards.

## Main text

*Apple chlorotic leaf spot virus* (ACLSV) and *Cherry green ring mottle virus* (CGRMV) have been detected worldwide and display a broad host range on pome and stone fruit trees [1]. However, as ACLSV is present in infected trees at a low concentration [2], and the two viral infections are also normally latent in some stone fruits [2-4], a sensitive and effective system is needed to detect ACLSV and CGRMV in stone fruits. Multiple viral infections are common in stone fruit trees [5,6]. Field surveys of peach viruses showed that some peach trees were infected with both ACLSV and CGRMV in China (unpublished data). Recently, three articles have reported plant virus detection using multiple RT-qPCR assays [7-9]. Therefore, we initiated this study to develop a method to determine the absolute copy numbers of ACLSV and CGRMV genomes in peach tissues, and to evaluate a duplex SYBR Green I-based RT-qPCR assay for the detection of ACLSV and CGRMV in a single reaction.

A total of 99 samples from leaf, branch bark, and flowers of peach infected with ALCSV and/or CGRMV and 34 leaf samples that showed mosaic symptoms were collected in China in 2012. Total RNAs were extracted from the tissue samples using the RNAprep Pure Plant Kit protocol (Tiangene, Beijing, China). A spectrophotometer (NanoDrop Technologies, USA) was used to quantify the RNA samples and determine their quality (an A_260_/A_280_ ratio between 1.9 and 2.1, and an A_260_/A_230_ ratio greater than 2.0).

Primer pairs AC84F/AC84R and CG94F/CG94R (Table 1) were used for normal PCR and predicted to amplify parts of the coat protein (CP) gene fragment (genomic locations 6,735-7,512 and 7,306-8235, respectively) of ACLSV and CGRMV, respectively. Each amplified DNA fragment was purified using a PCR purification kit (Axygen, Hangzhou, China) and inserted into the pGEM-T vector (Promega, USA). Purified recombinant plasmid DNA was linearized by restriction enzyme cleavage before in vitro transcription. Positive-strand RNA was transcribed using the RiboMAX Large Scale RNA Production Systems-T7 Kit (Promega, Madison, WI, USA). A RNA purification protocol (Promega) was used to remove the DNA template.

**Table 1:** Primer sequences and amplicon characteristics for PCRs

**PCR type**	**Name**	**Locus description**	**Primer Sequence (5’-3’)**	**Product size (bp)**	**Product TM (°C)**	**RT-qPCR Efficiency (%)**	**R**^**2**^	**Concentration (nM)**
qPCR^a^	AC62F	Coat protein	AAATACCCGGAGCTGATGTTTG	138	79.40 ± 0.24	97.0	0.9989	250
	AC62R		CTTCGCCTCATTTTCACTCTTTG					250
	CG732F	Coat protein	CAATTCAGGAGACGAACCCAG	181	81.44 ± 0.37	101.0	0.9939	250
	CG732R		TTCCCGACCATCTTTGTTTTG					250
	RPIIF	RNA polymerase subunit	TGAAGCATACACCTATGATGATGAAG	128	79.41 ± 0.29	93.6	0.9996	250
	RPIIR		CTTTGACAGCACCAGTAGATTCC					250
	UBQ10F	Ubiquitin 10	AAGGCTAAGATCCAAGACAAAGAG	146	84.36 ± 0.12	98.5	0.9958	500
	UBQ10R		CCACGAAGACGAAGCACTAAG					500
nPCR^b^	AC84F	Coat protein^c^	TCTGCAAGAGAATTTCAGTT	777	\	\	\	\
	AC84R		GTCTACAGGCTATTTATTATAAG		\	\	\	\
	CG94F	Coat protein^d^	CCTCATTCACATAGCTTAGGTTT	929	\	\	\	\
	CG94R		ACTTTAGCTTCGCCCCGTG		\	\	\	\

^a^quantitative PCR; ^b^normal PCR; ^c^genomic location 6735–7512, GenBank accession JN634761; ^d^genomic location 7306–8235, GenBank accession JX501671.

The sequences of primers used in the RT-qPCR assay are listed in Table 1. Both sets of primers were tested against each other and other major peach viruses by RT-PCR, and all of the results were negative (data not shown). This indicated that both sets of primers are highly specific for the viral sequences from which they were designed.

Two internal control genes, *RNA Polymerase subunit II* (*RPII*) and *Ubiquitin 10* (*UBQ10*) (peach EST database accession numbers TC1717 and TC2782, respectively), were used to evaluate the RT-qPCR assays as *UBQ10* and *RPII* are abundantly and constantly transcribed in all peach samples [10]. RNA extraction errors can be eliminated through the use of data analysis by the quantification of *UBQ10* and *RPII* expression.

**Figure 1:**
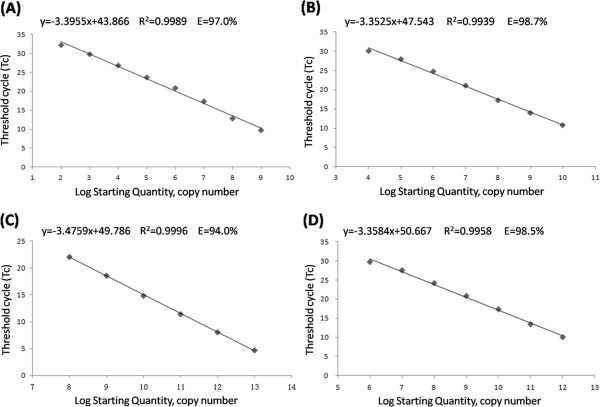
**Standard curves for SYBR Green I-based real-time RT-PCR amplification of standard ACLSV, CGRMV, *RPII *and *UBQ10 *RNA with specific primer pairs (see Table**1**).** Amplification plots showing the testing in duplicate of a 10-fold dilution series containing **(A)** standard ACLSV RNA from 1.08 × 10^9^ to 1.08 × 10^2^ template copies/reaction, **(B)** standard CGRMV RNA from 1.13 × 10^10^ to 1.13 × 10^4^ template copies/reaction, **(C)** standard *RPII* RNA from 2.09 × 10^13^ to 2.09 × 10^8^ template copies/reaction, and **(D)** standard *UBQ10* RNA from 1.90 × 10^12^ to 1.90 × 10^6^ template copies/reaction.

RNA templates for standard curves of the four genes were generated using the in vitro transcription method described by Zhang et al. in 2008 [11]. The purified RNA was quantified using the NanoDrop ND-1000 spectrophotometer (NanoDrop Technologies) and diluted 10-fold before use. One-step RT-qPCR reactions were performed using the GoTaq® 1-Step RT-qPCR System (Promega) according to manufacturer’s instructions. The final concentration of the AC62F, AC62R, CG732F, CG732R, RPIIF and RPIIR primers was 50 nM, whereas UBQ10F and UBQ10R were used at 100 nM. All of these concentrations had good levels of amplification efficiency (Figure 1).

The analytical sensitivities or detection limits of single RT-qPCR assays were determined by amplifying sequential 10-fold dilutions of quantified standard RNA. Four RNA standard curves were generated with primers and templates using the protocol described above (Figure 1). The standard curves for ACLSV, CGRMV, *RPII*, and *UBQ10* covered a linear range of eight, six, six and seven orders of magnitude (Figure 1A-D, respectively). The slopes and the correlation coefficients (R^2^) of the standard curves for ACLSV, CGRMV, *RPII* and *UBQ10* were suitable, as shown as Figure 1. This finding indicated that both the target RNAs in infected peach tissue and the reference RNAs could be quantified by these assays. This assay system proved to be highly sensitive, and could be used to detect ACLSV starting with as little as 10^2^ copies, and 10^4^ copies at CGRMV.

Single RT-qPCR was used to quantify genomic RNA copies of ACLSV and CGRMV and mRNA copies of *RPII* and *UBQ10* in different peach tissues. *RPII* and *UBQ10* mRNAs were quantified by this method to ensure that the assay system was reproducible. The number of copies per 100 ng of total RNA was given in Table 2. The coefficient of variation (CV) of the *RPII* and *UBQ10* assays (Table 1) showed a lower relative standard deviation, which suggested the RNA extraction and RT-qPCR methods were optimal for the quantification of ACLSV and CGRMV. This finding also showed that, as housekeeping genes, *RPII* and *UBQ10* were stably expressed genes across all of the tissues examined [10]. In the ACLSV assay, the copy numbers were 1.12 × 10^6^ ± 1.82 × 10^5^ in leaf, 2.14 × 10^6^ ± 5.13 × 10^5^ in bark and 1.38 × 10^7^ ± 3.31 × 10^6^ in the flower. In the CGRMV assay, the copy numbers were 4.07 × 10^8^ ± 4.17 × 10^7^ in leaf, 5.62 × 10^8^ ± 6.92 × 10^7^ in bark and 2.51 × 10^8^ ± 6.92 × 10^7^ in the flower. All data suggested that the absolute copy numbers of the ACLSV genome in leaf, bark and flower tissues of peach were lower than those of CGRMV. This result confirmed previous speculation regarding the low titer of ACLSV in stone fruit trees, and could to some extent explain the reason for the phenomenon of latent infection by ACLSV in stone fruits [2,3]. CGRMV has a relatively higher titer in bark, which was similar to the results found for *Citrus tristeza virus* in different citrus tissues [12], whereas the copy number of ACLSV is highest in flowers.

**Table 2:** ACLSV and CGRMV genomic RNA copy numbers in three peach tissues

	**Ct value (X ± S.D.)**^**a**^** [Number of copies (X ± S.E.)]**^**b**^			
	**ACLSV**	**CGRMV**	**RPII**	**UBQ10**
Leaf	23.32 ± 0.24^c^ [1.12 × 10^6^ ± 1.82 × 10^5^]	18.66 ± 0.23^f^ [4.07 × 10^8^ ± 4.17 × 10^7^]	17.16 ± 0.23^i^ [2.45 × 10^9^ ± 2.64 × 10^8^]	18.67 ± 0.18^l^ [3.39 × 10^9^ ± 3.02 × 10^8^]
Bark	22.38 ± 0.36^d^ [2.14 × 10^6^ ± 5.13 × 10^5^]	18.18 ± 0.30^g^ [5.62 × 10^8^ ± 6.92 × 10^7^]	17.46 ± 0.44^j^ [2.04 × 10^9^ ± 4.16 × 10^8^]	19.02 ± 0.30^m^ [2.68 × 10^9^ ± 3.89 × 10^8^]
Flower	19.61 ± 0.35^e^ [1.38 × 10^7^ ± 3.31 × 10^6^]	19.37 ± 0.40^h^ [2.51 × 10^8^ ± 6.92 × 10^7^]	17.76 ± 0.49^k^ [1.68 × 10^9^ ± 3.78 × 10^8^]	19.90 ± 0.41^n^ [1.47 × 10^9^ ± 2.91 × 10^8^]

^a^Average threshold cycle (Ct) and standard deviation (S.D.) obtained from 33 different samples.

^b^Average number of RNA copies (X) per sample and standard error (S.E.).

^c-n^Coefficient of variation (CV%) between assays: c = 1.02, d = 1.59, e = 1.76, f = 0.80, g = 1.59, h = 1.76, i = 0.70, j = 1.35, k = 1.51, l = 0.57, m = 0.95, n = 1.33.

**Figure 2:**
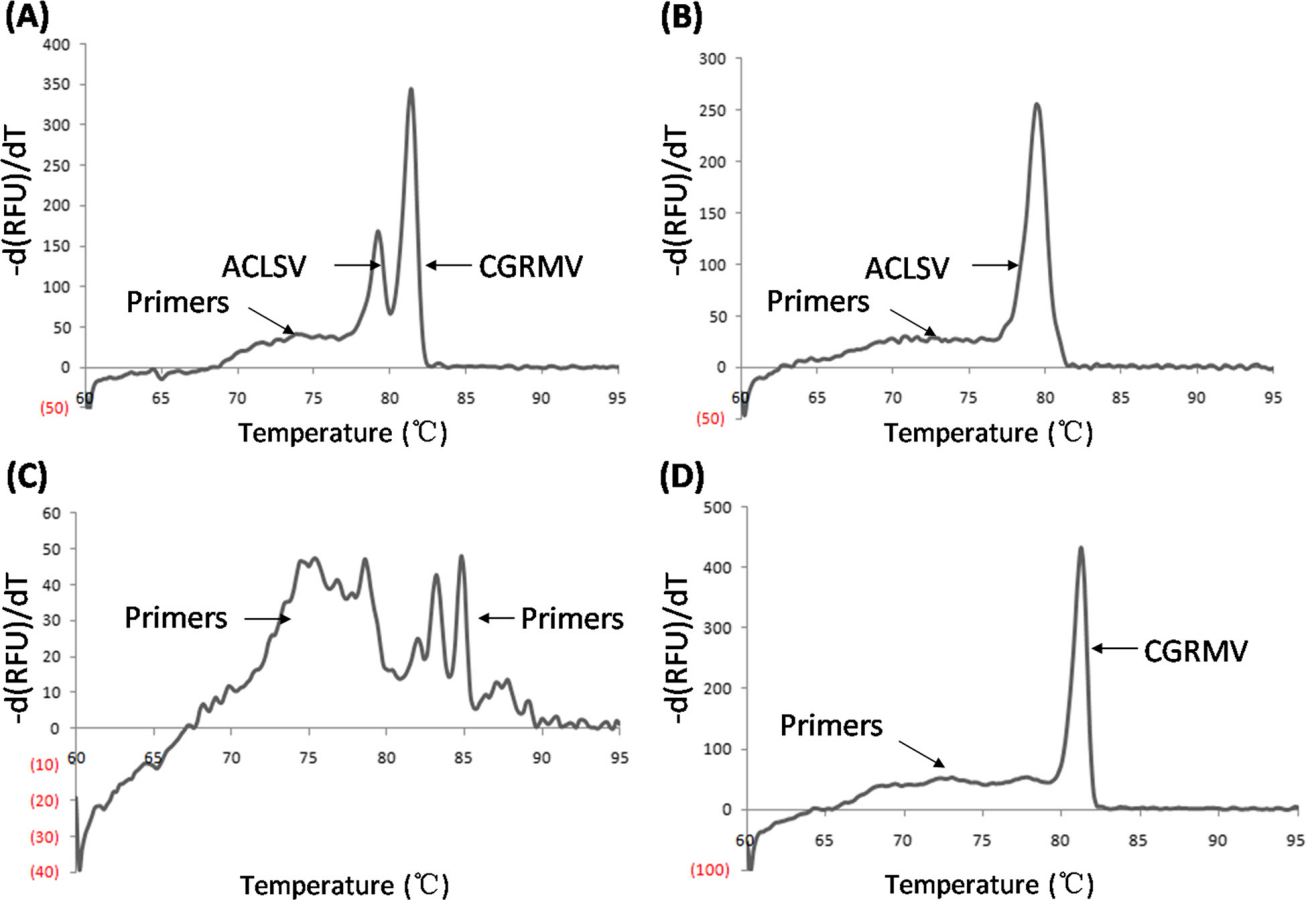
**Melting curve analysis for duplex RT-qPCR (A), single RT-qPCR (B and D), and no template RT-qPCR (C) as the negative control tested for ACLSCV and/or CGRMV.** Figure 2**C** shows the melting curve assay for primer pairs AC62F/AC62R and CG732F/CG732R; RNA from healthy peach tissue was used as a template.

A duplex SYBR Green-I RT-qPCR assay was developed to address the observation that peach trees are often infected by both ACLSV and CGRMV (unpublished data). As shown in Figure 2A, ACLSV and CGRMV could be discriminated in a duplex RT-qPCR reaction by melting curve analysis of the specific amplification products from the single RT-qPCR reactions. Specific melt peaks for ACLSV (T = 79.2°C) and CGRMV (T = 81.4°C) were obtained from the duplex RT-qPCR (Figure 2A). Also, the amplification products observed in the duplex RT-qPCR reaction were amplified using single RT-qPCR assays and had nearly identical melting peaks: 79.6°C for ACLSV and 81.2°C for CGRMV (Figures 2B and D). It can be seen from Figure 2A that the -d(RFU)/dT values for the ACLSV- and CGRMV-specific DNA fragments in the duplex RT-qPCR assay were similar to those in single RT-qPCR assays (Figures 2B and D), which indicated that the duplex assay can be used for the simultaneous detection of ACLSV and CGRMV. Healthy peach RNA (hpRNA) was used as the template in negative control (NC) reactions with the primer pairs AC62F/AC62R and CG732F/CG732R (Figure 2C). From this plot, the -d(RFU)/dT value of the primers was lower than 50, which was much lower than that of ACLSV or CGRMV, and showed a similar value to that shown in Figures 2A, B and D. The melting curve of the NC reactions with hpRNA and AC62F/AC62R or CG732F/CG732R primers resulted in a similar curve to that shown in Figure 2C (data not shown). These results suggested that primer dimers did not affect the PCR assays, and also showed that both of the primer pairs used for ACLSV and CGRMV detection were highly specific. We collected 34 field peach samples showing mosaic symptoms that were suspected to be infected with ACLSV and/or CGRMV from three sites in China (Table 3). ACLSV was detected in eight samples by RT-PCR and in ten samples by single and duplex RT-qPCR; five samples were CGRMV-positive by RT-PCR and six samples by single and duplex RT-qPCR. This result showed that single and duplex RT-qPCR assays are more sensitive than normal RT-PCR, and as a stable and effective detection system, the duplex RT-qPCR assay can be used to screen putatively infected peach trees in field.

**Table 3:** Results of RT-PCR, single RT-qPCR and duplex RT-qPCR detection of samples from different places infected with ALCSV and/or CGRMV

**No.**	**Location**	**RT-PCR**		**Single RT-qPCR**		**Duplex RT-qPCR**	
		**ACLSV**	**CGRMV**	**ACLSV**	**CGRMV**	**ACLSV**	**CGRMV**
1	Qingdao		+		+		+
2	Qingdao						
3	Qingdao			+		+	
4	Qingdao						
5	Qingdao						
6	Qingdao	+	+	+	+	+	+
7	Qingdao						
8	Qingdao				+		+
9	Qingdao						
10	Qingdao	+		+		+	
11	Qingdao						
12	Qingdao			+		+	
13	Qingdao						
14	Shijiazhuang	+		+		+	
15	Shijiazhuang						
16	Shijiazhuang	+		+		+	
17	Shijiazhuang						
18	Shijiazhuang		+		+		+
19	Shijiazhuang						
20	Shijiazhuang						
21	Shijiazhuang						
22	Shijiazhuang						
23	Taian	+		+		+	
24	Taian						
25	Taian						
26	Taian						
27	Taian						
28	Taian	+	+	+	+	+	+
29	Taian						
30	Taian	+		+		+	
31	Taian						
32	Taian						
33	Taian						
34	Taian	+	+	+	+	+	+
Total		8	5	10	6	10	6

Recently TaqMan-based multiplex RT-qPCR assays were used to detect viruses in tobacco, grapevine and rice [7-9]. SYBR Green-I multiplex RT-qPCR assays were developed for the simultaneous detection and quantification of animal viruses [13,14], and demonstrated that this strategy provides a reliable method for the detection and differentiation of nucleic acid targets. It also showed that multiple SYBR Green I-based RT-qPCR assays can retain a high level of sensitivity required for detection. Here, we described the detection of plant viruses using SYBR Green-I RT-qPCR assays, which have the advantages of economical and rapid identification of desired target genes. The duplex RT-qPCR assay and quantification of ACLSV and CGRMV titers in infected peach trees will provide a new method for the reproducible, sensitive and rapid detection of ACLSV and CGRMV. This will help to provide new insights into the biology of ACLSV and CGRMV that are necessary for disease control.

## Competing interests

The authors declare that they have no competing interests.

## Authors’ contributions

ZZ designed the research, participated in the sequence alignment, analyzed data and drafted the manuscript. YY designed primers for the RT-qPCR assays, and collected virus samples. ZXZ carried out the optimization of the RT-qPCR assays, contributed to the design of the study, primer design, sample collection, statistical analysis and designing the duplex RT-qPCR protocol. PBL and YXM extracted RNAs from peach tissues. All authors read and approved the final manuscript.
